# Association between Injections and HIV Incidence

**DOI:** 10.1371/journal.pmed.0020139

**Published:** 2005-05-31

**Authors:** Naveed Zafar Janjua, Khabir Ahmad, Arshad Altaf, Mohammad Imran Khan, Hasan Bin Hamza

**Affiliations:** Aga Khan UniversityKarachiPakistan

The article by Lopman and colleagues [1] has created more controversy than it's cured. Their conclusion that unsafe injections “do not play a major role in the transmission of HIV in rural Zimbabwe” cannot be based on the data they have presented. There are many major problems with their conclusions.

First, they did not assess at all whether injections received by the participants in the study were safe or unsafe. All they assessed was whether participants had received an injection or had been pricked by a needle during the past three years. The possibility that most of the injections in the setting were “safe” cannot be ruled out, and the data presented did not in any way address the issue of an association between “unsafe injection” practices and HIV infection.

Second, the recall period used by the investigators was too long. Cognizant of this problem, the World Health Organization has been proposing a three-month recall period for assessing frequency of injections. Although this recall period is 12 times shorter than the one used by the authors, we found in our studies in Pakistan that, even then, people had great difficulties recalling injections. It must be mentioned that injections are very frequent procedures in Pakistan [2].

Third, it seems that aside from injections, the investigators have included only data from sexual histories in their study; hence, other potential sources of exposure such as minor and major surgical procedures, dental instrumentation, and tattooing or other traditional practices involving scarification have been missed.

Fourth, it is not clear how “needle prick” was defined. Solid needles are also considered needles, but injury caused by these is less likely to transmit HIV than that caused by hollow bore needles.

Fifth, the authors have failed to quantify exposure. The risk of contracting HIV increases as the number of unsafe injections increases [3]. The incidence of disease among people who received one injection during follow-up compared with those who received 20 injections would clearly be different. This relationship has been clearly seen in the case of hepatitis C infection [4].

Sixth, the authors recommend that policymakers “should concentrate more on trying to prevent infection from unsafe sex” than on injections. But they have failed to assess whether the sex was unsafe or otherwise.

Further, we believe that even if the methodology is considered absolutely flawless, the current conclusion can only apply to a particular population and geographic area because the proportion of disease attributable to various exposures depends on the relative distribution of exposures in the population. For example, it is argued that in India (with the world's second largest HIV/AIDS population, more than 5 million) the HIV epidemic started as a result of high-risk sexual behaviors, but the number of injections per person is high and reuse of syringes in the health-care sector is widespread (N. K. Arora, personal communication). Therefore, unsafe medical injections have the potential to propagate this epidemic. Also, injections transmit many other pathogens like hepatitis B virus and hepatitis C virus. The infections that they cause have a very high morbidity and mortality. Hence, the need and urgency of intervention to decrease the overuse of injections and improve the safety of desired injections should not be questioned. Each country should make appropriate allocation of resources according to its own needs.
